# How Does the Chinese Government Select and Funding High-Level Talents? An Empirical Study Based on the Resumes of Talents

**DOI:** 10.3389/fpsyg.2021.687447

**Published:** 2021-09-17

**Authors:** Fan Wu, Jialin Su, Jingjing Zeng

**Affiliations:** ^1^School of Public policy and Management, Guangxi University, Nanning, China; ^2^School of Public Administration, Zhongnan University of Economics and Law, Wuhan, China

**Keywords:** talent selection, talent funding, high-level talents, resume, western China

## Abstract

Based on the textual data of the resumes of 499 high-level talents, this study attempts to explore the factors affecting the selection and funding of high-level talents in western China. From the empirical analysis, we found that (1) the western Chinese government tends to favor the young and native talents, with a high initial academic degree (the degree obtained before working) and final academic degree (the highest degree obtained); (2) the talents with more experience, higher education, national talent titles, and participation in national projects are more likely to receive higher levels of funding; (3) it is easier for talents in universities and research institutes to be entitled as high-level talents and to gain funding than those in enterprises; and (4) talents in the fields of medicine, agronomy, and basic sciences are more likely to be entitled as high-level talents than those in other professional fields.

## Key Points

- Collected the resume-related textual data of high-level talents and built a database of high-level talents in western China.
- Analyzed the influence of factors on the selection and funding of high-level talents based on the resumes of high-level talents in western China.
- Compared the selection and funding of high-level talents in different institutions and professional fields.

## Introduction

In China, the selection and funding of high-end talents have always been the effective means for regions to complement the tactic of “innovation-driven development.” The talent strategy of China has been the focus of the domestic and foreign academic circles (Zweig, 2006; Zhao and Leng, 2019) and aroused heated discussions in recent years (Wang, 2011; Niu and Zhou, 2012; Zhou et al., 2018). Since the inception of the People's Republic of China, from the “Academicians Program” headed by the Chinese Academy of Sciences (CAS, 1955) and the Chinese Academy of Engineering (CAE, 1994) to the national key high-level talent selection programs such as the “Thousand Talents Program” (TTP, 2008) and “Ten Thousand Talents Program” (TTTP, 2012), and high-level talent funding programs such as the “National Science Fund for Distinguished Young Scholars” (NSFDYS, 1994) and the “Chang Jiang Scholars Program” (CJSP, 1998), it has formed a complete selection and funding system for high-level talents, which is conducive to the cultivation and selection of the outstanding talents in China. Besides, local governments in China have launched a series of talent and funding programs, such as the “Phoenix Program” in Beijing, the “Hongyan Program” in Chongqing, and the “Bagui Scholars” in Guangxi. However, in the underdeveloped areas of western China, the difficulty in attracting, retaining, and cultivating talents has increasingly become the primary issue to be solved in the strategic adjustment of talents. Therefore, this study explored what factors should be highlighted in the selection and funding of high-level talents for the underdeveloped regions in China.

The existing research mainly analyzes talent selection from the perspective of the enterprise and examines the impacts of the knowledge, skills, and motivation of talents on the talent selection based on the “competency model” (McClelland, 1973; McLagan, 1980; Boyatzis, 1982). The research objects are mainly the corporate talents (Boyatzis, 1982; Bueno and Tubbs, 2004; Morrison, 2007), whereas few studies explore the selection of talents by local governments. Therefore, it lacks research on talent selection led by governments to promote regional scientific and technological innovation capacities. In terms of research methods, most research adopted the questionnaire survey (Chong, 2013; Skorková, 2016), but this method can hardly avoid measurement errors caused by subjective answers to some extent.

In this regard, this study included the high-level talents selected by the government as the research objects and explored the factors affecting the selection and funding of high-level talents in western China through the textual data of the resumes of 499 high-level talents. This study has the following three marginal contributions: First, it collected the resume-related textual data of high-level talents for the first time and built the database of high-level talents in western China. Second, based on the textual data of 499 resumes of high-level talents in western China, it empirically analyzed the influence of individual factors on the selection and funding of high-level talents and attempted to compare the selection and funding of high-level talents in different institutions and professional fields. Third, the results of this study can provide a scientific basis for the selection, funding, and cultivation of talents in remote areas, as well as suggestions for the academic career planning and development of high-level talents.

The remaining part of this article is organized as follows: the “Literature review and research hypotheses” section reviews the related literature and develops research hypotheses. The “Research design” section involves the design of the research, the interpretation of the research data sources, and main variables. The empirical results and discussion follow in the “Research results” section. The “Further analyses: Heterogeneity test of institution and professional field” section reports the heterogeneity test of different institutions and professional fields. Finally, the “Discussion and conclusion” section summarizes and concludes.

## Literature Review and Research HYPOTHESES

The factors affecting the selection and funding of high-level talents mainly involve three aspects, namely, personality, human capital, and cumulative advantage.

### Personality and Talent Selection

Personality is an important factor for success (Feist, 1998).

Studies have found that demographic characteristics such as age, gender, and birthplace will have an impact on the selection and funding of high-level talents. Human creativity is bound up with age (Manniche and Falk, 1957; Simonton, 1988; Baffes and Vamvakidis, 2011). At present, there are two explanations for the relationship between age and scientific research output of talents: First, some scholars reckon that the best creative age for the scientific elites presents the unimodal shape. Taking the age distribution of Nobel laureates as an example, physics laureates are younger than scientists in the field of economics (Bjork, 2019). Studies have found that the age of the highest professional achievement of top scientists gradually shifts back, and the peak age of scientific research is also moving backward (Jones and Weinberg, 2011). Furthermore, the Chinese scholars have also found that the recipients of major national science funds have an older trend (Han and Li, 2016). Second, others deem that the best creative age of most ordinary researchers shows the bimodal or the inverted U-shaped model. After the academic value created by an individual reaches its peak in the mature period, academic creativity will slowly decline (Aksnes et al., 2011).

From the perspective of the growth characteristics of scientific and technological talents, the performance of scientific and technological talents has phase characteristics (Zhang et al., 2018). Talent funding projects run through the entire life cycle of the career development of scientific and technological talents and promote the cultivation of scientific research ability and career development of scientific and technological talents, exchanges and cooperation of international talents, etc. (Zhang et al., 2017). Moreover, funding can significantly improve scientific productivity, maintain participation in the science department, and affect “brain drain” (Ganguli, 2017). The current research has also found that there are some management difficulties such as uneven distribution of disciplines, excessive proportion of introduced talents, obvious gender differences, and regional preferences in the funding of the Outstanding Youth Science Fund and the National Outstanding Youth Science Fund, which restrict the economic and technological development of underdevelopment regions in China (Zhao et al., 2017). In the youth talent funding programs in China, the proportion of women is lower than that of men (Wang and Ren, 2013), that is, men are more likely to obtain funding for scientific research projects than women (Symonds et al., 2006).

From the perspective of the preference of the talents for geographical location, some scholars pointed out that due to the resource endowment advantages of developed cities, the Chinese scientists are more willing to move to the eastern region, resulting in the spatial distribution of the Chinese scientists showing more in the east and less in the west (Wu et al., 2003; Abel and Deitz, 2009). From the perspective of the institution, talents are more willing to work in areas with many colleges and universities, which can provide numerous opportunities for the development of talents. The study found that the funders of the NSFDYS (“Jieqing”) tend to gather in the southeast region, and there is a significant input of talent from universities. Therefore, the high-level youth talents in China are more likely to work in “prestigious universities and research institutes” (Rui, 2017). However, in recent years, it has shown a trend that more and more talents choose to work in western China (Harvey, 2014), which may be attributed to policy factors, family factors, and the pursuit of self-actualization of the individuals (Ioannidis, 2004; Soon, 2010a,b). Therefore, this study proposes the following hypotheses:

Hypothesis 1a: The older the talents, the more likely they are to be selected as high-level talents and to receive a higher level of academic recognition and gain more funding.
Hypothesis 1b: Compared to female talents, male talents are more likely to be selected as high-level talents and to receive a higher level of academic recognition and gain more funding.
Hypothesis 1c: Compared to talents in other regions, the native Guangxi talents are more likely to be selected as high-level talents and to receive a higher level of academic recognition and gain more funding.

### Human Capital and Talent Selection

Human capital is an important factor affecting talent selection. Individual knowledge, education, skills, and health are the key elements that constitute human capital (Schultz, 1961). The human capital possessed by individuals can be assessed on the basis of their educational background, work experience, and identifiable skills (Harrison et al., 2001). The factors, including educational background, work experience, and professional skills (Tharenou et al., 1994; Kirchmeyer, 1998), have more impacts on individual career success. Besides, the factors, such as education level, education quality, job involvement, work experience, and length of service, are positively related to career success (Ng et al., 2005; Ballout, 2007). Therefore, education level, education quality, and work experience are the important factors that affect career success.

Empirical studies have revealed that the factors, such as education, intelligence, work competitiveness, and technical skills, are highly correlated with individual career success (Keeton, 1993). At different life stages, these factors have different impacts on career success (Seibert et al., 1999). Educational background, major, graduate school, and work experience have positive effects on the career success of American executives (Judge et al., 1999). In particular, educational background, graduate school, and post-doctoral experience have positive impacts on the growth of scientific researchers, especially on those who receive a strict formal education or obtain a doctorate from a prestigious school (Zuckerman and Jonathan, 1976). The experience of being selected and funded is part of career success. By measuring the human capital of talents, it can effectively predict the possibility of being selected and funded. Therefore, this study proposes the following hypotheses:

Hypothesis 2a: The higher education level the talents have, the more likely they are to be selected as high-level talents and to receive a higher level of academic recognition and gain more funding.
Hypothesis 2b: Compared to talents from the non-prestigious schools, those from prestigious schools are more likely to be selected as high-level talents and to receive a higher level of academic recognition and gain more funding.
Hypothesis 2c: The longer the length of service in Guangxi, the more likely talents are to be selected as high-level talent and to receive a higher level of academic recognition and gain more funding.
Hypothesis 2d: Compared to talents without skills certificates, those with skills certificates are more likely to be selected as high-level talents and to receive a higher level of academic recognition and gain more funding.

### Cumulative Advantage and Talent Selection

The growth of an outstanding scientist fully reflects the “cumulative advantage” (Merton, 1968, 1973, 1988). The accumulation of advantages is mainly reflected in the honorary titles obtained by individual talents, published articles and studies, overseas study experience, and funding. Even in many cases, these indicators have become an important basis for the Chinese local governments to select talents.

In China, the “TTP,” “Hundred Talents Program,” “TTTP,” “CJSP,” “Outstanding Youth,” and other titles reflect the academic qualification and scientific research influence of talents to some extent (Niu and Zhou, 2012), and the obtainment of these titles can significantly enhance the achievement motivation of scientific and technological talents (Wang et al., 2020). For academic institutions, the introduction of talents with the title of “TTP” can lead to strong academic productivity and influence (Zhao and Ye, 2014). In fact, scientists with the title of “Hundred Talents Program” have comprehensively improved various institutes of the Chinese Academy of Sciences, in terms of the quantity and quality of the international academic articles (Li and Ha, 2017).

In addition, many scholars believe that talent funding shows obvious features of funding concentration, that is, the Matthew effect (Leydesdorff et al., 2019). Recent studies have also found that talent funding projects are increasingly concentrated in the hands of a few researchers (Bol et al., 2018; Liao, 2021). However, some studies have also found that the other funded high-end scientific and technological talents in China have increasingly close cooperation at home and abroad in the scientific research output, and the effect is significant (Liu and Yang, 2017). However, the output of the Chinese Natural Science Youth Projects is mostly the foreign journals, the completion rate fluctuates greatly, and the conversion rate of scientific and technological achievements is not high (Rui, 2017). Moreover, the amount of funding received by most research institutions is negatively correlated with the output of the Chinese scientific research in the institution (Yin et al., 2018), i.e., there is no absolute positive correlation between the funding received by talents and the output of individual scientific research. Many researchers in the world use project funding to promote their own career development. Generally speaking, the pass rate is not high. In particular, early career researchers (ECRs) have a greater failure rate, which will lead to the loss of ECR talents (de Winde et al., 2021). From the individual level, researchers need to face reality and maintain hope (Holdsworth, 2020); from the institutional level, the government optimizes funding selection, making it more legal and scientific and boosting the career development of the research personnel (Besselaar and Arensbergen, 2013).

As a result, with the increase in government funding for talents, more attention has been paid to talent funding research, focusing on the status of funded projects and the analysis of the research output of project funders and their individual characteristics. Social network analysis and data analysis are used as the main methods in these studies, while resume analysis, questionnaire survey, and other attempts are rarely used. In data processing, descriptive statistical analysis dominates, and there are not many processing methods such as regression analysis. In general, there are many digable themes in the field of talent funding, such as the individual growth law of funded recipients and the performance results of funded projects, enriching the research content of this field. Therefore, this study proposes the following hypotheses:

Hypothesis 3a: The higher the titles talents have, the more likely they are to be selected as high-level talents and to receive a higher level of academic recognition and gain more funding.
Hypothesis 3b: Compared to talents without overseas study experience, those with overseas study experience are more likely to be selected as high-level talents and to receive a higher level of academic recognition and gain more funding.
Hypothesis 3c: Compared to talents without titles, those with titles are more likely to be selected as high-level talents and to receive a higher level of academic recognition and gain more funding.
Hypothesis 3d: Compared to talents without scientific research achievements, those with scientific research achievements are more likely to be selected as high-level talents and to receive a higher level of academic recognition and gain more funding.

## Research Design

### Data Sources

#### Data Collection

The data source of this study is based on the *Academic Recognition Measures for High-level Talents in Guangxi Zhuang Autonomous Region* (*No. 36 [2017] of Guibanfa*). With the help of the Department of Human Resources and Social Security of Guangxi Zhuang Autonomous Region, a list of 499 high-level talents (only 336 of them were selected as the high-level talents) were obtained, and the resume data of these talents were collected in the later stage. The title of high-level talents discussed in this study is the provincial-level title, which specifically refers to the high-level innovative talents working in Guangxi. Based on the performance, contribution, industry, and social recognition of the talents, the title is divided into five levels from high to low, namely, A, B, C, D, and E.

#### Data Cleaning

By browsing the authoritative websites and querying the china national knowledge infrastructure (CNKI) database, the data matching and data improvement were carried out on the collected list of 499 talents. Then, combined with the field investigation and telephonic interviews, we established a basic resume database of 499 talents and figured out whether these talents had obtained the title of high-level talents. Overall, we collected the basic information, such as the age, gender, academic background, hometown (native of Guangxi or not), educational background, length of service in Guangxi, graduation institution, overseas study experience, scientific research results, and title and funding status of the talents.

### Variable Measurement

The dependent variables are as follows: “whether to obtain the title of high-level talent,” “the level of academic recognition,” and “the amount of funding received.”

Whether to obtain the title of high-level talent refers to whether candidates were selected as high-level talents. The level of academic recognition refers to the different levels (as mentioned earlier, there are five levels, i.e., A, B, C, D, and E) given to the selected 499 talents. The amount of funding received includes post-allowance, housing allowance, and team allowance, all of which are continuous variables.

The independent variables are as follows: “personality,” “human capital,” and “cumulative advantage.”

Personality includes these measurement indicators, such as age, gender, and hometown (native of Guangxi or not). Human capital includes education level, graduate school, work experience, and skills certificate. Education level includes two variables, namely, initial academic degree (the degree obtained before working) and final academic degree (the highest degree obtained). Cumulative advantage includes professional qualification, overseas study experience, talent title, and national-level project. For more details, refer to Table 1.

**Table 1:** Value nd descriptive statistics of each variable.

**Variables**	**Descriptions**
**Dependent variables**	
Title of high-level talent	No = 0, yes = 1
Level of academic recognition	Level A = 5, Level B = 4, Level C = 3, Level D = 2, Level E = 1^a^
Post-allowance	It refers to the relevant funding provided to talents for their scientific research. Please see the actual data.
Housing allowance	It refers to the funding provided by the local government to solve the housing issue of talents. Please see the actual data.
Team allowance	It refers to necessary scientific research funding and support for talents to conduct research in a team. Please see the actual data.
**Independent variables**	
**Personality**	
Age	2020—Year of birth
Hometown	No = 0, yes = 1
Gender	Female = 0, male = 1
**Human capital**	
Initial academic degree	Below junior college = 0, junior college = 1, bachelor = 2, master = 3, doctor = 4, post-doctor = 5
Final academic degree	Below junior college = 0, junior college = 1, bachelor = 2, master = 3, doctor = 4, post-doctor = 5
Key university	Non-211 or non-985 = 0, 211 = 1, 985 = 2, 211 and 985 = 3^b^
Length of service in Guangxi	Deadline of work contract—Start time of work contract
Skills certificate	No = 0, yes = 1
**Cumulative advantage**	
Professional qualification	No = 0; junior = 1; intermediate = 2; senior = 3; professor = 4^c^
Overseas talent	No = 0, Yes = 1^d^
Number of national-level titles	It refers to the number of honorary titles obtained by participating in national-level talent selection programs. Please see the actual data.
Number of national-level funding	It refers to the number of funded projects obtained by participating in the selection of national-level talent funding projects. Please see the actual data.
Number of provincial-level titles	It refers to the number of honorary titles obtained by participating in provincial-level talent selection programs. Please see the actual data.

a *This study reverse–coded the level of academic recognition of high-level talents, the higher the level, the greater the code, which can make the results of our research more explanatory*.

b *The number 985 refers to the universities included in Project 985, which is a project implemented by the Chinese government to build several world–class universities and some internationally renowned high-level research universities. There are 39 universities included in Project 985; 211 refers to the universities included in Project 211, which aims to build about 100 key universities and several key disciplines in the twenty–first century. There are 114 universities included in Project 211*.

c *In China, the professional qualification refers to the technical and academic level and work capacity of professional and technical personnel. It reflects the technical level and working capacity of professional and technical personnel. It is mainly divided into junior, intermediate, and senior levels*.

d *It refers to the overseas talent with the title of the national “TTP,” the “Hundred Talents Program” launched by the Chinese Academy of Sciences, and the “Hundred Overseas Talents” launched by Guangxi. These three titles are provided to the high-level overseas talents with overseas study or work experience, which are the key introduction and training objects of the state, institutions, or local governments*.

## Research Results

### Model Design

As “talent selection” is an ordered variable, we employed logit regression and ordinary least square (OLS) regression to analyze the factors affecting this variable, based on the control of the clustering effect of the location of the institution, professional field, and institution.

The dependent variables included here “whether to obtain the title of high-level talent,” “level of academic recognition,” and “the amount of funding received” denote the regression coefficient corresponding to each independent variable, indicating the fixed effect. The three fixed effects, namely, the location of the institution, professional field, and institution denote the coefficient corresponding to the fixed effects, indicating the random error.

### Model Test

This study cross-checked the predictive effect of the model, and the specific method is as follows: K-fold Cross Validation was applied to cross-check the model, and the samples were divided into 10 groups. Finally, the correctly classified variable value prediction of the training data of the model was 89.18%. The correctly classified variable value prediction obtained by training the model with the cross-check method was 87.58%, the area under the curve (AUC) value of all samples was 0.9267, and the AUC value of the test samples was 0.9057. It can be observed that the fit of all data training models and cross-check training models was high, so the prediction effect of this model was good.

### Dependent and Independent Variables

Table 2 describes the statistical characteristics of the dependent and independent variables. It can be observed that the average value of the dependent variable “whether to obtain the title of high-level talent” is 0.68, and the average value of the variable “level of academic recognition” is 1.64, indicating that 70% of the 499 talents obtained the title of high-level talent, and the “level of academic recognition” of most talents is D or E. Among the three types of allowances, the highest is “housing allowance.” In terms of the “personality,” the average value of “age” is 48.5 years old, the average value of “hometown” is 0.48, and the average value of “gender” is 0.83, that is, among the 499 talents, half of them are from Guangxi and male talent is predominant. The average values of variables “initial academic degree,” “final academic degree,” “key university,” and “length of service in Guangxi” are 1.37, 3.08, 0.57, and 16.77, respectively, indicating that among the candidates, most of them have a bachelor's degree or above in the “initial academic degree,” master's degree in the “final academic degree,” whereas they did not graduate from prestigious schools, and have worked in Guangxi for a long time. In terms of the “cumulative advantage,” the average value of the variable “professional qualification” is 3.70, that is, most talents have senior titles or they are professors; the average value of “overseas talents or not” is 0.16, that is, only a few of them have obtained the titles of “Thousand Talents Plan,” “Hundred Talents Plan,” and the “Hundred Overseas Talents” launched by Guangxi. The average values of “national-level title,” “national-level project,” and “provincial-level title” are 0.42, 0.06, and 0.69, respectively. That is to say, only a few talents have participated in the national-level projects. More results from the OLS estimation are reported in Appendix B (Supplementary Material).

**Table 2:** Descriptive statistics of related variables.

	***N***	**Mean**	***SD***	**Min**	**Max**
**Dependent variables**					
**Level 1**					
Title of high-level talent	499	0.673	0.469	0	1
Level of academic recognition	499	1.641	0.897	1	5
Post-allowance	499	5.813	9.219	0	100
Housing allowance	499	39.495	33.064	0	200
Team allowance	499	17.816	51.397	0	1,000
**Independent variables**					
**Personality**					
Age	499	48.533	8.467	27	80
Hometown	499	0.483	0.5	0	1
Gender	499	0.83	0.376	0	1
**Human capital**					
Initial academic degree	499	1.373	0.915	0	2
Final academic degree	499	3.082	1.676	0	5
Key university	499	0.569	1.034	0	3
Length of service in Guangxi	499	16.768	14.466	0	56
Skills certificate	499	0.391	0.488	0	1
**Cumulative advantage**					
Professional qualification	499	3.697	0.775	0	4
Overseas talent	499	0.16	0.367	0	1
Number of national-level titles	499	0.415	0.551	0	3
Number of national-level funding	499	0.06	0.254	0	2
Number of provincial-level titles	499	0.687	0.569	0	3
**Fixed effects**					
**Level 2**					
Location	499	3.567	2.28	1	12
Professional field	499	9.317	5.804	1	21
Institution	499	3.85	1.551	1	5

### Hypothesis Testing

Hypothesis testing of the influence of “personality” on talent selection (Table 3). As shown in Table 3, young people are more likely to obtain the “title of high-level talent,” while the elderly are more likely to obtain a higher “level of academic recognition.” Thus, Hypothesis 1a is partially supported. With regard to the testing of Hypothesis 1b, the regression results indicate that “gender” does not have a significant impact on talent selection. Therefore, this hypothesis is rejected. Hypothesis 1c is also partially supported. The regression results reveal that talents from Guangxi are more likely to obtain the “title of high-level talent,” but the “hometown” does not affect the “level of academic recognition” of talents.

**Table 3:** Regression results of related variables.

	**Title of high-level talent**		**Level of academic recognition**	
	**Coefficient (SE)**	**Odds ratio**	**Coefficient (SE)**	**Odds ratio**
**Personality**				
Age	−0.10*** (−3.55)	0.90***	0.14*** (7.39)	1.15***
Hometown	1.39*** (3.97)	4.01***	−0.03 (−0.14)	0.97
Gender	0.39 (0.95)	1.47	0.31 (0.96)	1.36
**Human capital**				
Initial academic degree	1.04*** (4.67)	2.83***	0.19 (1.16)	1.21
Final academic degree	0.94*** (6.79)	2.57***	0.75*** (6.84)	2.12***
Key university	0.33* (1.67)	1.40*	0.23** (2.32)	1.26**
Length of service in Guangxi	−0.03** (−1.98)	0.97**	0.00 (0.01)	1.00
Skills certificate	0.18 (0.51)	1.19	0.53** (2.30)	1.70**
**Cumulative advantage**				
Professional qualification	−0.12 (−0.45)	0.89	0.55** (2.50)	1.73**
Overseas talent	1.35 (1.24)	3.85	−0.81** (−2.29)	0.45**
Number of national-level titles	0.68** (2.02)	1.97**	0.38 (1.52)	1.46
Number of national-level funding	3.15*** (2.72)	23.38***	1.25*** (3.34)	3.49***
Number of provincial-level titles	0.37 (1.20)	1.45	−0.96*** (−4.40)	0.38***
**Fixed effects**				
Location	0.12* (1.66)	1.13*	0.09* (1.7998)	1.09*
Professional field	0.01 (0.49)	1.01	0.07*** (3.45)	1.07***
Institution	−0.31*** (−2.68)	0.73***	−0.19** (−2.28)	0.83**
**Thresholds**				
cut1			12.29*** (9.77)	12.29***
cut2			14.22*** (10.87)	14.22***
cut3			16.00*** (11.83)	16.00***
cut4			19.71*** (11.49)	19.71***
*N*	499	499	499	499
Pseudo *R*^2^	0.53	0.53	0.28	0.28

* *p < 0.10*,

** *p < 0.05*,

*** *and p < 0.01*.

Hypothesis testing of the influence of “human capital” on talent selection. As the regression results show, if talents have a higher “initial academic degree” and “final academic degree,” they will be more likely to obtain the “title of high-level talent.” Besides, the “final academic degree” further affects the “level of academic recognition.” Thus, Hypothesis 2a is almost supported. With respect to the testing of Hypothesis 2b, the regression results indicate that talents graduating from prestigious schools, such as universities included in Project 985 or Project 211, are more likely to obtain the “title of high-level talent.” With regard to the testing of Hypothesis 2c, the regression results manifest that this hypothesis is rejected at the 5% level of significance, that is, under the same conditions, it is easier for talents with a shorter “length of service in Guangxi” to obtain the “title of high-level talent.” In terms of the testing of Hypothesis 2d, the regression results show that the “skills certificate” has no significant impact on the obtainment of talents of the “title of high-level talent.” However, under the same conditions, a skills certificate improves the “level of academic recognition.”

Hypothesis testing of the impact of “cumulative advantage” on talent selection. Except for Hypothesis 3b, others are supported in varying degrees. As the regression results show, talents with high “professional qualification” are more likely to obtain the high “level of academic recognition,” that is, Hypothesis 3a is supported. However, for Hypothesis 3b, the regression results manifest that, under the same conditions, those with “overseas study experience” have no advantage in gaining the “title of high-level talent,” and the “overseas study experience” is not conducive to the obtainment of high “level of academic recognition.” In terms of the testing of the Hypothesis 3c, the regression results show that it is easier for the talents with “national-level title” to be selected as the “high-level talent” than those with “provincial-level title,” and the more the talents with “provincial-level title” obtain, the less they are likely to gain high “level of academic recognition.” With respect to the testing of Hypothesis 3d, the regression results indicate that the participation of “national-level projects” has a significant positive impact on talent selection.

The regression analysis was performed with “post-allowance,” “housing allowance,” and “team allowance” as dependent variables (Table 4). As shown in Table 4, “age” has statistically significant effects on “post-allowance,” “housing allowance,” and “team allowance,” but the effects of “hometown” and “gender” on them are not significant. This indicates that the older the talents, the more likely they obtain allowance. “Human capital” as a whole has a significant influence on “post-allowance,” “housing allowance,” and “team allowance,” indicating that the funding policies in the underdeveloped regions favor talents with higher education level and from famous universities. The “length of service in Guangxi” negatively affects “housing allowance” and “team allowance,” indicating that the shorter the “length of service in Guangxi,” the easier the talents obtain funding. This may be related to the preference of funding policies in the underdeveloped regions, hoping to attract more young talents through funding policies. The obtainment of “skills certificates” also makes it easier to get “post-allowance” and “housing allowance.” As for the “cumulative advantage,” “national-level title” and “national-level project” have significant effects on the three types of allowances, indicating that the funding policies of the underdeveloped regions do not favor talents with “professional qualifications” and “overseas study experience” but prefer talents who have obtained “national-level titles” and participated in “national-level projects.” It is worth mentioning that talents with “provincial-level titles” have no advantage in obtaining relevant funding.

**Table 4:** Regression results of different funding.

	**Post-allowance**	**Housing allowance**	**Team allowance**
	**Coefficient (SE)**	**Coefficient (SE)**	**Coefficient (SE)**
**Personality**			
Age	0.36*** (6.27)	1.44*** (7.07)	1.94*** (5.54)
Hometown	−0.72 (−0.96)	−2.09 (−0.79)	−6.79 (−1.48)
Gender	0.67 (0.72)	2.41 (0.73)	0.10 (0.02)
**Human capital**			
Initial academic degree	0.55 (0.99)	2.99 (1.53)	6.16* (1.82)
Final academic degree	1.31*** (4.38)	5.33*** (5.03)	4.34** (2.37)
Key university	0.50 (1.37)	2.80** (2.14)	−0.27 (−0.12)
Length of service in Guangxi	0.04 (1.20)	−0.23** (−2.02)	−0.41** (−2.06)
Skills certificate	1.48* (1.89)	7.25*** (2.61)	6.17 (1.29)
**Cumulative advantage**			
Professional qualification	0.54 (1.04)	1.63 (0.89)	0.16 (0.05)
Overseas talent	−0.88 (−0.76)	−0.20 (−0.05)	0.43 (0.06)
Number of national-level title	2.50*** (3.34)	10.67*** (4.01)	20.72*** (4.51)
Number of national-level funding	3.46** (2.41)	15.54*** (3.05)	19.28** (2.19)
Number of provincial-level title	−2.73*** (−3.92)	−8.86*** (−3.59)	−5.85 (−1.37)
**Fixed effects**			
Location1	0.39** (2.53)	−0.19 (−0.34)	0.60 (0.63)
Professional field1	0.15** (2.34)	0.60*** (2.66)	0.31 (0.79)
Institution1	0.04 (0.14)	−1.12 (−1.23)	−0.07 (−0.04)
_cons	−22.22*** (−8.09)	−58.11*** (−5.95)	−101.55*** (−6.03)
*N*	499	499	499
Pseudo *R*^2^			

* *p < 0.10*,

** *p < 0.05*,

*** *and p < 0.01*.

## Further Analyses: Heterogeneity Test of Institution and Professional Field

### The Impact of Different Institutions on Talent Selection

We explored the influence of the different “institutions” on the obtainment of the “title of high-level talent,” the “level of academic recognition,” and the “amount of funding received” (Table 5). The results show that compared to the talents in “enterprises,” the talents in “scientific research institutes” are more likely to obtain “title of high-level talents,” gain high “level of academic recognition,” and get “post-allowance.” Compared to talents in “enterprises,” those in “universities” have no significant advantage in obtaining the “title of high-level talent.” However, it is easier for talents in “universities” to obtain post and housing allowances than those in “enterprises.” We provided three possible explanations for this difference. First, the scientific research conditions and environment of “universities” and “scientific research institutes” are better than those of “enterprises,” so talents in “universities” and “scientific research institutes” are more likely to achieve good scientific research results that are expected of government funding. Second, the various funds given by the state or local government to “universities” and “scientific research institutes” are more abundant than those of “enterprises.” In addition, local “universities” and “scientific research institutes” are the gathering places of high-level talents, so they need more talent funding and training than enterprises. We also found that talents in “other public institutions” have no advantage in obtaining the “title of high-level talent” compared to those in “enterprises,” indicating that under the same conditions, talents in “enterprises” are more likely to be selected as high-level talents than those in “other public institutions.”

**Table 5:** The impact of different institutions on talent selection and funding.

	**Logit regression**				**OLS regression**		
	**Title of high–level talent**		**Level of academic recognition**		**Post-allowance**	**Housing allowance**	**Team allowance**
	**Coefficient** **(SE)**	**Odds** **ratio**	**Coefficient** **(SE)**	**Odds** **ratio**	**Coefficient** **(SE)**	**Coefficient** **(SE)**	**Coefficient** **(SE)**
**Institution**							
Enterprise	0.00 (.)	0.00	0.00 (.)	0.00	0.00 (.)	0.00 (.)	0.00 (.)
Other public institutions	−1.27* (−1.76)	0.28*	0.35 (0.53)	1.42	3.22 (1.06)	10.89 (0.99)	2.75 (0.16)
Hospital	0.15 (0.36)	1.16	0.13 (0.35)	1.14	0.58 (0.32)	−1.28 (−0.20)	0.97 (0.10)
Scientific research institute	0.62* (1.92)	1.85*	0.52* (1.82)	1.68*	2.31* (1.70)	6.72 (1.38)	7.84 (1.03)
University	0.40 (1.62)	1.49	0.40* (1.65)	1.49*	2.82** (2.58)	7.55* (1.92)	9.38 (1.53)
_cons	0.42** (2.03)	1.52**	–	–	3.78*** (4.03)	34.11*** (10.13)	11.25** (2.14)
**Thresholds**							
cut1			0.68*** (3.30)	1.97***			
cut2			1.87*** (8.38)	6.49***			
cut3			3.24*** (11.60)	25.53***			
cut4			6.55*** (6.42)	699.24***			
*N*	499		499		499	499	499
Pseudo *R*^2^	0.0165		0.0040				

* *p < 0.10*,

** *p < 0.05*,

*** *and p < 0.01*.

### The Impact of Different Professional Fields on Talent Selection

We examined the influence of different “professional fields” on the obtainment of the “title of high-level talent,” the “level of academic recognition,” and the “amount of funding received” (Table 6). The results indicated that compared to the talents in “other professional fields,” those engaged in “agronomy,” “engineering,” “medicine,” “liberal arts,” and “science” are more likely to be selected as high-level talents. Specifically, the number of talents in “science” who are selected as high-level talents is 23.17 times than those in “other professional fields,” and the number of talents in “liberal arts” who obtained the high “level of academic recognition” is 62.52 times than those in “other professional fields.” In terms of “team allowance,” it is easier for talents in “medicine” and “science” to obtain “team allowance” than those in “agronomy,” “engineering,” “liberal arts,” and “other professional fields.” As far as post and housing allowances are concerned, talents in “liberal arts” gain the most post and housing allowances. It may be due to the following reasons: First, “medicine” and “science” are the basic research disciplines, with an emphasis on experiments and the formation of scientific research teams (deB Beaver and Rosen, 1978, 1979). Some studies have pointed out that experimental scientists are more cooperative than theoretical scientists (Gordon, 1980), especially in the field of basic research, which needs more cooperation than other non-basic fields (Newman, 2001). Therefore, basic sciences such as “medicine” and “science” need more funding, especially team funding, to better organize scientific research teams and conduct research. Second, “liberal arts” highlight theories. Compared to basic disciplines such as “science,” “engineering,” “medicine,” and “agronomy,” “liberal arts” have lower requirements for scientific research facilities, office conditions, and scientific research team building, but they have higher requirements for the platform.

**Table 6:** The impact of different professional fields on talent selection and funding.

	**Logit regression**				**OLS regression**		
	**Title of high-level talent**		**Level of academic recognition**		**Post-allowance**	**Housing allowance**	**Team allowance**
	**Coefficient** **(SE)**	**Odds** **ratio**	**Coefficient** **(SE)**	**Odds** **ratio**	**Coefficient** **(SE)**	**Coefficient** **(SE)**	**Coefficient** **(SE)**
**Professional fields**							
Other professional fields	0.00 (.)		0.00 (.)		0.00 (.)	0.00 (.)	0.00 (.)
Agronomy	2.52*** (5.22)	12.47***	3.31*** (3.18)	27.29***	5.24*** (2.95)	15.57** (2.44)	14.61 (1.44)
Engineering	1.69*** (3.74)	8.12***	3.16*** (3.05)	18.80***	3.40** (1.98)	11.74* (1.91)	11.93 (1.22)
Medicine	2.09*** (4.67)	5.41***	2.93*** (2.83)	23.52***	5.02*** (2.99)	17.96*** (2.98)	24.75*** (2.59)
Liberal arts	1.34*** (2.62)	3.80***	4.14*** (3.93)	62.52***	9.34*** (4.63)	30.79*** (4.24)	10.52 (0.91)
Science	3.14*** (6.94)	23.17***	3.79*** (3.70)	44.21***	7.19*** (4.54)	25.58*** (4.50)	23.43*** (2.60)
_cons	−1.39*** (−3.51)	0.25***	–	–	0.40 (0.28)	20.90*** (4.10)	0.25 (0.03)
**Thresholds**							
cut1			3.67*** (3.63)	3.67***			
cut2			4.94*** (4.85)	4.94***			
cut3			6.34*** (6.14)	6.34***			
cut4			9.65*** (6.78)	9.65***			
*N*	499		499		499	499	499
Pseudo *R*^2^	0.1223		0.0516				

* *p < 0.10*,

** *p < 0.05*,

*** *and p < 0.01*.

## Discussion and Conclusion

This study analyzed the text of resumes of high-level talents in western China, explored the factors affecting the selection of high-level talents, and revealed the mechanism behind the selection of high-level talents in the underdeveloped regions in western China. The results indicated that as follows: (1) in terms of talent selection, the selection of high-level talents in the underdeveloped regions in China favors the young and native talents, with high academic qualifications and degrees from prestigious schools. Additionally, the talents who have obtained the national-level titles and participated in the national-level projects are more likely to be selected as high-level talents. However, those with provincial-level titles have no advantage in talent selection; (2) with respect to funding, the talents with old age, high academic qualifications, national-level titles, and experience in national-level projects are more likely to obtain high funding for post, team, and housing allowances. However, the funding policies of the underdeveloped regions favor the talents with a short length of service in Guangxi; and (3) based on the heterogeneity analysis, we found that it is easier for talents in universities and scientific research institutes to obtain the title of high-level talent and gain funding than those in enterprises; the talents in medical, agronomy, and basic sciences are more likely to obtain a high level of academic recognition than those in other professional fields. With respect to the allowances received, talents in medicine and science are more likely to receive higher team allowances than those in other professional fields, and talents in literal arts tend to receive higher post and housing allowances. Therefore, the selection criteria of high-level talents should be formulated according to the reality of the western regions.

This study can provide significant references for the formulation, implementation, and optimization of selection and training plans of high-level talents for the underdeveloped regions. First, age-related funding policies should be inclusive, and the selection of high-level talents should be reasonable in age distribution. We should not only attach importance to the selection, training, and funding of young talents but also give full play to the role of “cumulative advantage” in the career growth of middle-aged and elderly scientists and create conditions and opportunities for their second peak of scientific research creativity. Second, we should highlight the impacts of the non-traditional factors on the introduction of high-level talents, from relying solely on material support to providing both material and non-material supports. Third, local governments should formulate innovative talent incentive and security policies, strengthen and develop the endogenous motivation of talent incentive mechanism, and take into account the competitive relationship between foreign talents and local talents. Moreover, it is necessary to value the talents who have been working in Guangxi for a long time and have made great contributions to the region, while making use of the funding policies to attract young talents. Fourth, it is necessary to give play to the incentive role of the non-material support, as well as the guarantee function of the material support such as funding policies. The number of allowances for various professional fields should be allocated reasonably, and more post and team allowances should be given to talents in basic disciplines. The talents in liberal arts should gain more funding in housing. Fifth, the selection of talents should not only consider “academic qualifications,” “titles,” and “projects.” In other words, the selection and cultivation of talents in the underdeveloped regions should facilitate the local scientific and technological progress, the high-quality economic development, and the transformation and upgrading of industrial structure. Finally, it is necessary to improve the scientific research facilities of local universities and scientific research institutes and to create an excellent scientific research atmosphere, so as to provide sufficient space for talents to make use of their capacities.

This study has some limitations. First, this study mainly explored the individual factors affecting the selection of high-level talents in western China. Apart from these factors, there might be other influencing factors, such as the attractiveness of local talent policies, the level of regional competitiveness, and the organizational support. Second, the independent variables involved in this study may have hierarchical relationships, but we did not explore the relationship between the variables. Finally, this study did not discuss the team-level selection. Hence, future research can be improved in these aspects.

## Data Availability Statement

The raw data supporting the conclusions of this article will be made available by the authors, without undue reservation.

## Author Contributions

JZ and FW: conceptualization, methodology, software, validation, formal analysis, resources, writing-original draft preparation, writing-review and editing, visualization, supervision, project administration, and funding acquisition. JS: investigation. JZ: data curation. All authors contributed to the article and approved the submitted version.

## Funding

This research was financially supported by the project of the Philosophy and Social Science Planning Research of Guangxi (20FGL050), the fund project of the National Natural Science Foundation of China (Grant No. 71974203), the Zhongnan University of Economics and Law Fundamental Research Funds for the Central Universities (2722021BX021), and the Innovation and Talent Base for Income Distribution and Public Finance (B20084).

## Conflict of Interest

The authors declare that the research was conducted in the absence of any commercial or financial relationships that could be construed as a potential conflict of interest.

## Publisher's Note

All claims expressed in this article are solely those of the authors and do not necessarily represent those of their affiliated organizations, or those of the publisher, the editors and the reviewers. Any product that may be evaluated in this article, or claim that may be made by its manufacturer, is not guaranteed or endorsed by the publisher.
