# Supplementary material for: How Does the Chinese Government Select and Funding High-Level Talents? An Empirical Study Based on the Resumes of Talents
Source: Front Psychol. 2021 Sep 17;12:687447. doi: 10.3389/fpsyg.2021.687447 (PMC8484645; doi:10.3389/fpsyg.2021.687447)

**Appendix A-1**

**Appendix A-1 A detailed list of personnel inputs in the early stage of data collection for this study**

| **Serial number** | **Time** | **Research task** | **Participants** |
| --- | --- | --- | --- |
| 1 | 2018 | The original resume materials are sorted and classified; the name and ID information of the original resume materials are deleted | FanXuekai (Graduate of 2018)  DengMengyun(Graduateof 2016)  ZhuFangman(Graduate of 2017) |
| 2 | 2019 | Original resume data sorting and inputting to the computer | FanXuekai (Graduate of 2018)  Su Jialin (Graduate of 2018)  Liu Xiangling (graduate of 2018) |
| 3 | 2019 | 335 people CV database 47 field information supplemented and perfected | Fan Xuekai (Graduate of 2018)  Zhang Haile (Grade 2019 undergraduate)  Zhang Huashan (Graduate of 2019) |
| 4 | 2020 | Restore the name information of the original material; match the network data information according to the name | Zhang Haile (Grade 2019 undergraduate)  Lu Jingxian (Class of 2017 undergraduate)  Kaili Chen (graduate of 2019) |
| 5 | 2020 | Classification of initial and highest academic qualifications, and final data proofreading | Zhang Haile (Grade 2019 undergraduate)  Lu Jingxian (Class of 2017 undergraduate)  Su Jialin (Graduate of 2018)  Wu Fan (Author) |

**Appendix A-2 List of basic information about interviews in this research**

| **Serial number** | **Time** | **Interviewee and work unit** | **Purpose of interview** |
| --- | --- | --- | --- |
| 1 | 2019 | Miss Qin,  Guangxi Department of Human Resources and Social Security | Hometown and initial education of the personnel collecting missing data |
| 2 | 2019 | Miss Zhao,  Guangxi Department of Human Resources and Social Security | Hometown and job title of personnel collecting missing data |
| 3 | 2019 | Miss Deng,  Guangxi Subtropical Crops Research Institute | Hometown of the person who collected the missing data |
| 4 | 2019 | Miss Feng,  Guangxi Subtropical Crops Research Institute | Collect and improve personal resume data and job titles |
| 5 | 2019 | Mr. Zhang  Guangxi Medical University | Hometown of the person who collected the missing data |
| 6 | 2020 | Miss Zeng,  Guangxi Medical University | Hometown of the person who collected the missing data |
| 7 | 2020 | Miss Wei,  Guangxi University | Hometown of the person who collected the missing data |
| 8 | 2020 | Miss Li ,  Guilin University of Technology | Hometown of the person who collected the missing data |

**Appendix B**

**Table A-3**  **Robustness Check by OLS estimation**

|  | **Title of high-level talent** | **Level of academic recognition** |
| --- | --- | --- |
| ***Personality*** |  |  |
| Age | -0.01*** | 0.04*** |
|  | (0.00) | (0.01) |
| Hometown | 0.15*** | -0.06 |
|  | (0.03) | (0.07) |
| Gender | 0.02 | 0.05 |
|  | (0.04) | (0.08) |
| ***Human capital*** |  |  |
| Initial academic degree | 0.15*** | 0.07 |
|  | (0.02) | (0.05) |
| Final academic degree | 0.13*** | 0.18*** |
|  | (0.01) | (0.03) |
| Key university | 0.03* | 0.07** |
|  | (0.02) | (0.03) |
| skills certificate | 0.02 | 0.17** |
|  | (0.03) | (0.07) |
| Length of service in Guangxi | -0.00** | -0.00 |
|  | (0.00) | (0.00) |
| ***Cumulative advantage*** |  |  |
| Professional qualification | -0.02 | 0.07 |
|  | (0.02) | (0.05) |
| Overseas talent | -0.02 | -0.18* |
|  | (0.05) | (0.10) |
|  |  |  |
| Number of national-level titles | 0.07** | 0.17** |
|  | (0.03) | (0.07) |
| Number of national-level funding | 0.24*** | 0.50*** |
|  | (0.06) | (0.13) |
| Number of provincial-level titles | 0.02 | -0.32*** |
|  | (0.03) | (0.06) |
| ***Fixed effects*** |  |  |
| location | 0.01** | 0.03** |
|  | (0.01) | (0.01) |
| Professional field | 0.00 | 0.02*** |
|  | (0.00) | (0.01) |
| Institution | -0.03*** | -0.03 |
|  | (0.01) | (0.02) |
| Constant | 0.53*** | -1.35*** |
|  | (0.12) | (0.25) |
| Observations | 499 | 499 |
| R-squared | 0.538 | 0.429 |

Standard errors in parentheses

*** p<0.01, ** p<0.05, * p<0.1

**Appendix C**


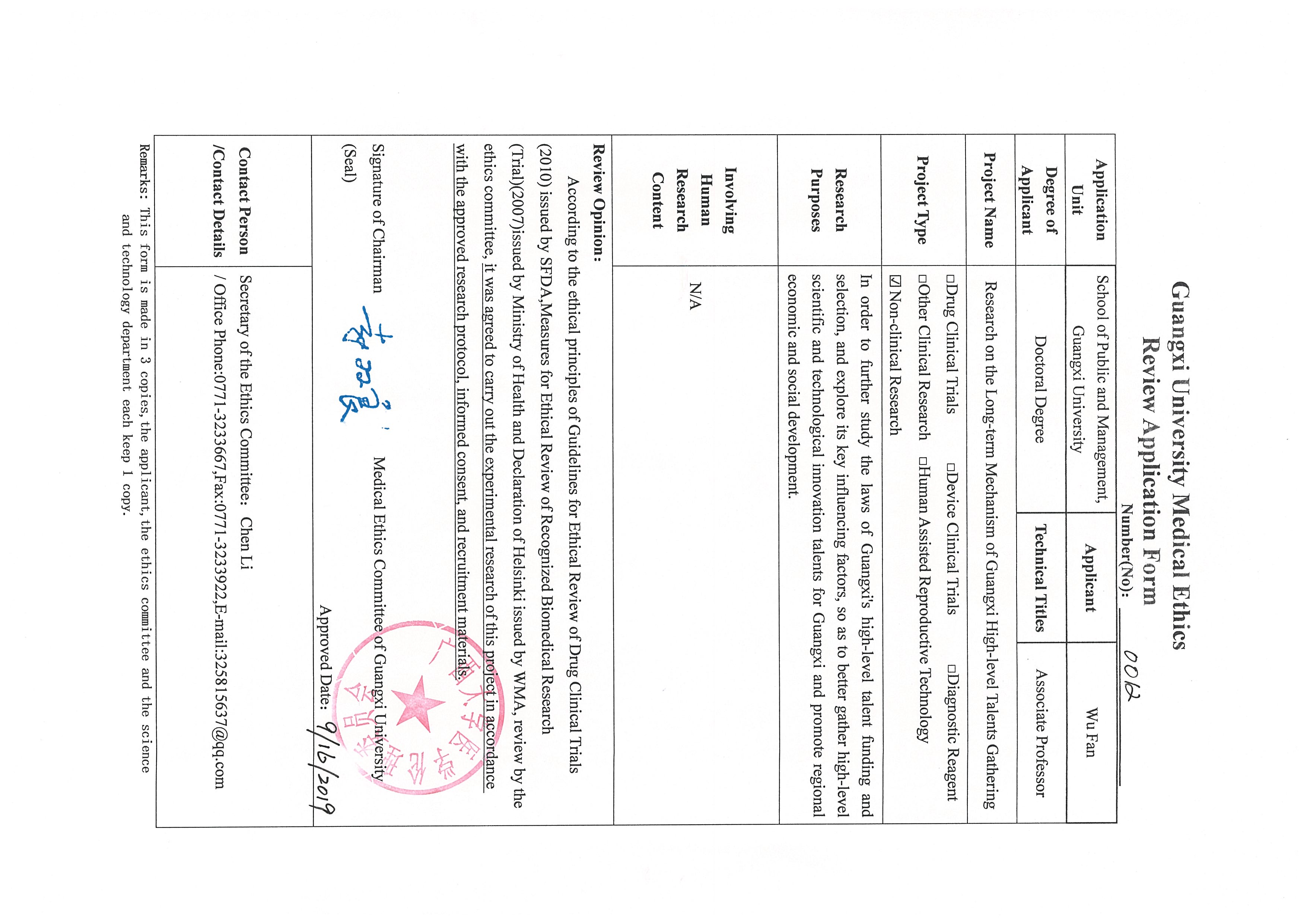

Supplement: Supplementary file 1 [file Data_Sheet_1.docx]
